# COVID-19 and Pediatric Rheumatology: A Comprehensive Study from a Leading Tertiary Center in Saudi Arabia

**DOI:** 10.1007/s44197-023-00142-z

**Published:** 2023-08-18

**Authors:** Jubran Alqanatish, Abdullah Almojali, Abdulmajeed Alfadhel, Areej Albelali, Amal Ahmed, Abdullah Alqahtani, Abdulrhman Alrasheed, Wafaa Alsewairi, Suliman Alghnam

**Affiliations:** 1 College of Medicine, King Saud Bin Abdulaziz University for Health Sciences, 14611 Riyadh, Saudi Arabia; 2 King Abdullah International Medical Research Center (KAIMRC), 14611 Riyadh, Saudi Arabia; 3 King Abdullah Specialized Children’s Hospital, King Abdulaziz Medical City (National Guard Health Affairs), 14611 Riyadh, Saudi Arabia

**Keywords:** SARS-CoV-2 infection, COVID-19, Rheumatic disease, Children, Adolescents, Hospitalization, Saudi Arabia

## Abstract

The Coronavirus disease 2019 (COVID-19) pandemic has emerged as a significant global health concern, impacting millions of individuals worldwide. However, there remains a notable gap in the literature regarding pediatric studies, specifically focusing on children with rheumatic diseases and the potential risk factors associated with COVID-19 contraction in this specific patient population. Patients with rheumatic diseases are often undergoing immunemodulator/immunosuppressant therapies, which can further complicate their immune system response to infections. This is a retrospective cohort study conducted at King Abdullah Specialized Children’s Hospital (KASCH), the largest tertiary care children’s hospital in Saudi Arabia. The aim was to investigate the rate, clinical manifestations, risk factors, and outcomes of COVID-19 infection in pediatric patients with rheumatic diseases. All rheumatology patients (< 19 years) who presented to the hospital as outpatients, inpatients, and/or ER visits during the period of March 2020 to March 2022 were reviewed for confirmed diagnosis of COVID-19. Among 482 patients included in this study, 126 (26.1%, 95% CI 21.8–31.1) had COVID-19 infection, and no factors were identified to increase the risk of contracting the virus. Fever (55.6%, *n* = 70) followed by respiratory symptoms (55.6%, *n* = 70) were the most common clinical manifestations, and around 30% of the patients were asymptomatic. Though most of the patients recovered without complications (97.6%, *n* = 123), mortality was reported in 3 patients (2.38%). The risk of hospitalization was almost 6 times higher in males (OR = 5.97), and higher in patients receiving t-DMARDs (OR = 17.53) or glucocorticoids (OR = 6.69). The study also revealed that vaccinated children were at lower risk of hospitalization due to COVID-19 than non-vaccinated children. The findings of this study help to identify the risk factors for COVID-19 among children with rheumatic diseases and provide insight into the impact of the pandemic on this group. Overall, while most cases were mild and resolved on their own, unvaccinated patients and those receiving t-DMARDs or glucocorticoids needs vigilant monitoring during the COVID-19 infection. Furthermore, we strongly advocate for the widespread promotion of COVID-19 vaccination among pediatric rheumatology patients as it significantly reduces their risk of COVID-19-related hospitalization.

## Introduction

The World Health Organization (WHO) declared the outbreak of acute respiratory infection that emerged in Wuhan, China in December 2019 as a public health emergency of international concern (PHEIC) in Feb 2020. The disease Corona Virus Infectious Disease-2019 (COVID-19) was caused by a novel beta-Coronavirus called SARS-CoV-2 virus. The rapid international spread of the disease lead WHO to declare the disease as a global pandemic on March 2020 [1, 2]. In addition of being a novel virus causing a highly contagious viral infection, leading to fatal acute respiratory distress syndrome (ARDS) with high mortality rate were among the key factors for the international fear and panic [1–3]. As of the time of writing this article (30 April 2023), 765,180,259 confirmed cases and 6,924,926 deaths have been reported globally [4]. Currently, after 3 years from the outbreak, the WHO international health regulation COVID-19 emergency committee decided to clear the pandemic in its 15th meeting held on May 2023 [5].

Since the early days of the pandemic, physicians and public health authorities conducted multiple research projects trying to identify risk factors and predictors of COVID-19 complications and mortality in their patient populations, especially those with compromised immune system. Patients with rheumatic diseases are one of the populations who may require special consideration as many of them are receiving immunomodulator and/or immunosuppressant therapies.

Many articles were published describing adult rheumatology patients with COVID-19 infections. One study showed that people with rheumatic disease were not at increased risk of developing severe respiratory complications as speculated [6]. Another study documented a statistically significant association between hospitalization of COVID-19 patients with steroid use (> 5 mg/day), previous history of lung diseases, and male gender [7]. In addition, elderly patients (> 65 years) were found to have an increased risk of hospitalization and complicated COVID-19 pneumonia [8]. Interestingly, no clear association was found between the risk of COVID19 or its complications in patients receiving biologics or conventional disease-modifying antirheumatic drugs (DMARDs) [7, 8].

On the other hand, pediatric population have been less affected by COVID-19 virus in terms of frequency and severity [9–11]. One large case series of 72,314 COVID-19 cases showed that only 2% of patients were children and adolescents [12]. Several factors were thought to be associated with this milder course of infection including the low incidence comorbidities [13], smoking, and travelling history [14] in children compared to adults. However, no clear reason was identified or confirmed to explain why children are less affected by this virus. What is unique to pediatrics is the development of multisystem inflammatory syndrome in children (MIS-C) which was first identified in April 2020, 2–4 weeks following severe SARS-CoV-2 infection [15, 16].

As this viral infection is usually mild and less frequent in children, the literature is deficient in pediatric studies in general, and this deficiency is more profound when it comes to pediatric rheumatology studies. To date, only one study was done in pediatrics with rheumatic and musculoskeletal diseases using three international registries for COVID-19 in pediatric rheumatology. It investigated the short-term outcomes of the infection in 609 patients (< 19 years) from 25 European & North American countries and concluded that SARS-CoV-2 infection is mild and has an overall good prognosis in this population [17].

Locally, multiple studies on COVID-19 in the pediatric population were conducted in Saudi Arabia. One study looked at a cohort of 742 children diagnosed with COVID-19, and outlined the clinical characteristics, diagnostic findings, and therapeutic interventions among this cohort [11]. Another study, reported on a cohort of 88 hospitalized COVID-19 patients in a multicenter study, while another multicenter study examined the clinical characteristics and outcome of Multisystem Inflammatory Syndrome, although none of the patients in all these studies had a primary rheumatological disease diagnosis [18, 19].

In addition to the limited availability of local studies regarding COVID19 infection in pediatric patients with rheumatic diseases, there is a significant gap in global research regarding the factors that may predispose this patient population to contracting the virus. Specifically, there is insufficient understanding of the impact of various medications, including conventional and targeted DMARDs and steroids. Therefore, the aim of this study is to investigate the epidemiology, clinical manifestations, risk factors, and outcomes of COVID-19 in pediatric patients with rheumatic diseases at King Abdullah Specialist Children’s Hospital. As a leading tertiary center in Saudi Arabia for childcare, this facility is uniquely positioned to contribute significant insights to the existing literature.

## Materials and Methods

This is a retrospective cohort study conducted at King Abdullah Specialized Children’s Hospital (KASCH), the largest tertiary care children’s hospital in Saudi Arabia. The pediatric rheumatology division at KASCH provides care to children with rheumatic disorders and variable multi-system inflammatory conditions.

Electronic medical records for patients with pediatric rheumatic diseases were identified through the Hospital Information System (BESTcare). The inclusion criteria for detailed medical record review included all rheumatology patients (< 19 years) who presented to the hospital as outpatients, inpatients, and/or ER visits during the period of March 2020 to March 2022 and had done at least one polymerase chain reaction (PCR) during this period. COVID-19 infection was defined as having a positive PCR test. Two pediatric rheumatology consultants screened the identified files, and patients with no specific underlying rheumatological diseases or those who did not do PCR were excluded.

Demographic data, primary rheumatic disease, medication history, and the clinical characteristics of COVID-19 infection were obtained from the patients’ records. Patients with MIS-C were defined as per the 2020 criteria adapted from the US Centers for Disease Control (CDC) [20]. The study was conducted in adherence with ethical policies. It was approved by the Institutional Review Board of King Abdullah International Medical Research Center (KAIMRC). The anonymity and confidentiality of the research subjects and their medical records was maintained.

The data were entered in Microsoft Excel 2019 and analyzed with the Statistical Package for the Social Sciences (SPSS), version 25 (IBM Corp., Armonk, NY, USA). The rate of COVID-19 infection in pediatric rheumatology patients was reported as percent with 95% CI. Categorical variables were presented by frequency and percentages, and continuous variables by mean and standard deviation. The effect of patients’ demographics and baseline characteristics on contracting COVID-19 infection was examined using the Pearson’s Chi-square test. In addition, the association between hospital admission of COVID-19 patients with their demographic variables was assessed by the Pearson’s Chi-square test or, for small sample size, the Fisher Exact Test. Hospitalization in COVID-19 patients was used as a dependent variable, and patients’ demographics as independent variables. A test with a *P* value of < 0.05 was considered significant.

## Results

**Fig. 1:**
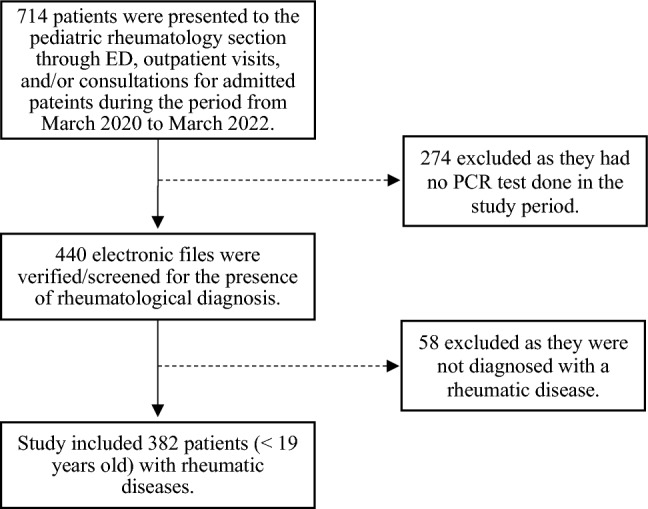
Flow chart visualizing the patient’s selection process. *ED* emergency department, *PCR* polymerase chain reaction

During the period from March 2020 to March 2022, a total of 714 patients presented to the pediatric rheumatology section at King Abdullah Specialized Children’s Hospital. Two hundred seventy-four patients had no PCR test in their files during that period, and another 58 patients were not yet diagnosed with a rheumatologic disease, resulting in a total of 332 excluded patients (Fig. 1).

The study was done on 482 pediatric rheumatology patients. The mean age was 13.2 ± 4.5 years, and the majority of them were adolescents (71%, *n* = 342). The percentage of female patients was slightly higher (54.8%, *n* = 264 vs. 45.2% *n* = 218), and their clinical characteristics are shown in Table 1. The most common primary disease was juvenile idiopathic arthritis (39.2%, *n* = 189) followed by acute rheumatic fever and rheumatic heart disease (29.5%, *n* = 142). Other diagnoses were autoinflammatory diseases (17.2%, *n* = 83) and multisystemic autoimmune disorders (14.1%, *n* = 68). Almost half of the patients received at least one dose of COVID-19 vaccine. Two hundred fourteen children (44.4%) were known to have other comorbidities; the most common were cardiac diseases in 79 children, followed by neurological disorders in 31 children, pulmonary in 28 children, gastrointestinal and liver diseases in 28 children and the remaining comorbidities were various including renal, behavioral, and psychiatric disorders.

**Table 1:** Patients’ demographics and clinical characteristics (*N* = 482), and comparison between patients who had COVID-19 infection (*n* = 126) and those who did not (*n* = 356)

Characteristic	Level	Total		Had COVID-19		Not infected		*P* value
		*n*	%	*n*	%	*n*	%	
Sex	Male	218	45.2	52	41.3	166	46.6	0.3
	Female	264	54.8	74	58.7	190	53.4	
Age groups	< 6 years	45	9.3	15	12.7	29	8.1	0.06
	6– < 12 years	95	19.7	17	13.5	78	21.9	
	12– < 19 years	342	71	93	73.8	249	69.9	
Comorbidities	No	268	55.6	69	54.8	199	55.9	0.83
	Yes	214	44.4	57	45.2	157	44.1	
On medications	No	398	82.6	24	19	60	16.9	0.58
	Yes	84	17.4	102	81	296	83	
c-DMARDs	No	296	61.4	80	63.5	216	60.7	0.58
	Yes	186	38.6	46	36.5	140	39.3	
t-DMARDs	No	272	56.4	70	55.6	202	56.7	0.82
	Yes	210	43.6	56	44.4	154	43.3	
Methotrexate	No	335	69.5	88	69.8	247	69.4	0.92
	Yes	147	30.5	38	30.2	109	30.6	
Hydroxychloroquine	No	450	93.4	118	93.7	332	93.3	0.88
	Yes	32	6.6	8	6.3	24	6.7	
Glucocorticoids	No	318	66	86	68.3	232	65.2	0.53
	Yes	164	34	40	31.7	124	34.8	
Adalimumab	No	414	85.9	111	88.1	303	85.1	0.4
	Yes	68	14.1	15	11.9	53	14.9	
Vaccinated	No	250	51.9	56	44.4	194	54.5	0.05
	Yes	232	48.1	70	55.6	162	45.5	
COVID-19 vaccine doses	One dose	36	15.5	10	14.3	26	16	0.73
	Two doses	196	84.5	60	85.7	136	84	

*P* values were calculated using the Pearson’s chi-square test. *COVID*-*19* Coronavirus disease 2019, *c*-*DMARDs* conventional disease-modifying antirheumatic drugs, *t-DMARDs* targeted disease-modifying antirheumatic drugs

Among the 482 pediatric rheumatology patients in this study, one hundred twenty-six patients (26.1%, 95 CI 21.8–31.1%) had COVID-19 infection. Comparison between the patients who had COVID19 infection (*n* = 126) and those who did not (*n* = 356) is shown in Table 1. There was no statistically significant difference in gender, age groups, pre-existing comorbidities, COVID19 vaccine doses, nor using conventional or targeted DMARDs.

**Fig. 2:**
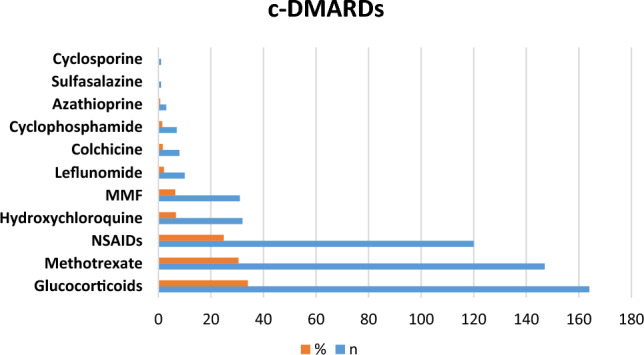
Conventional disease-modifying antirheumatic drugs (c-DMARDs). *MMF* mycophenolate mofetil, *NSAIDs* non-steroidal anti-inflammatory drugs

**Fig. 3:**
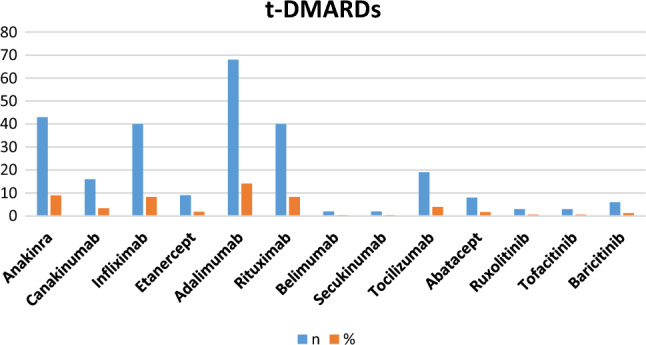
Targeted disease-modifying antirheumatic drugs (t-DMARDs)

Most of the patients were on regular medications for their primary rheumatic disease (82.6%, *n* = 392). Conventional disease-modifying antirheumatic drugs (c-DMARDs) were used by 186 children (38.6%), and targeted DMARDs (t-DMARDs) were used in 210 patients (43.6%). Figure 2 shows that 164 patients were using corticosteroid, representing the most frequently used c-DMARDs, followed by Methotrexate (147 patients), and NSAIDs (120 patients). With regard to t-DMARDs, Tumor Necrosis Factor inhibitors (anti-TNF) was the most frequently used class (117 patients), followed by IL-1 inhibitors (59 patients), and anti-B-cell drugs (42 patients). Figure 3 shows that Adalimumab was the most frequently used targeted drug (68 patients), followed by Anakinra (43 patients), then Infliximab (40 patients) and Rituximab (40 patients).

As shown in Table 2, Almost 30% (*n* = 37) of patients who got COVID-19 were asymptomatic during the time of infection. Fever was the most frequent clinical manifestation (55.6%, *n* = 70), followed by respiratory symptoms (50.8%, *n* = 64) and malaise (17.5%, *n* = 22). A total of 19 patients were hospitalized, representing 15.1% of all positive cases. Overall, most of the patients recovered from their COVID19 infection without complications (84.9%, *n* = 107), and mortality was reported in 3 patients (2.38%).

**Table 2:** Clinical presentation of COVID-19 in pediatric rheumatology patients (*n* = 126)

Characteristic	Level	*n*	%
COVID-19 symptoms at onset	No	37	29.40
	Yes	89	70.60
Symptoms	Fever	70	55.56
	Respiratory symptoms	64	50.79
	Malaise	22	17.46
	Headache	17	13.49
	Flare of the disease	12	9.52
	GI symptoms	11	8.73
	Myalgia	6	4.76
	Loss of smell	5	3.97
	Skin rash	5	3.97
	Arthralgia	5	3.97
	Odynophagia	2	1.59
Hospitalization	No	107	84.92
	Yes	19	15.08
Complications	None	104	82.54
	MIS-C	11	8.73
	Respiratory failure	6	4.76
	Myocarditis	4	3.17
	Acute liver injury	3	2.38
	Acute kidney injury	3	2.38
Outcome	Recovery	123	97.62
	Death	3	2.38

*COVID*-*19* coronavirus disease 2019, *MIS*-*C* multisystem inflammatory syndrome in children

Table 3 shows the association between hospitalization with the study variables among patients with COVID19 infection (*n* = 126). There is a statistically significant association between hospitalization with male gender (*p* = 0.009) and young age (*p* < 0.001). In addition, exposure to t-DMARDs or glucocorticoids was significantly associated with hospitalization (94.3% vs. 5.7%, *p* < 0.001), (78.9% vs. 21.1%, *p* < 0.001), respectively.

**Table 3:** Association between hospitalization and study variables among patients with COVID-19 infection (*n* = 126)

Characteristic	Level	Not hospitalized		Required hospitalization		*P* value
		*n*	%	*n*	%	
Sex	Male	39	36.4%	13	68.4%	0.009*
	Female	68	63.6%	6	31.6%	
Age groups^†^	< 6 years	5	4.7%	11	57.9%	0.000*
	6– < 12 years	14	13.1%	3	15.8%	
	12– < 19 years	88	82.2%	5	26.3%	
c-DMARDs	No	65	60.7%	15	78.9%	0.129
	Yes	42	39.3%	4	21.1%	
t-DMARDs	No	69	64.5%	1	5.3%	0.000*
	Yes	38	35.5%	18	94.7%	
Vaccinated	No	39	36.4%	17	89.5%	0.000*
	Yes	68	63.6%	2	10.5%	
Glucocorticoids	No	82	76.6%	4	21.1%	0.000*
	Yes	25	23.4%	15	78.9%	
Comorbidities	No	62	57.9%	7	36.8%	0.089
	Yes	45	42.1%	12	63.2%	

*P* values were calculated using the Pearson’s chi-square test except for age groups

*COVID*-*19* coronavirus disease 2019, *c*-*DMARDs* conventional disease-modifying antirheumatic drugs, *t-DMARDs* targeted disease-modifying antirheumatic drugs

^†^*P* value was calculated by the Fisher’s Exact Test for age groups

*Significant *P* value (< 0.05).

Logistic regression was carried out to assess the predictors of hospitalization (the dependent variable), as shown in Table 4. The risk of hospitalization is almost 6 times higher in males (OR = 5.97, 95% CI [1.44–24.71]). Vaccinated patients were at lower risk to be admitted due to COVID-19 in comparison with those who did not received the vaccine (OR = 0.09, 95% CI [0.02–0.654]). In addition, receiving t-DMARDs (OR = 17.53, 95% CI [1.8–165.9]) or glucocorticoids (OR = 6.69, 95% CI [1.46–30.64]) were predictors of hospitalization.

**Table 4:** Predictors of hospitalization among patients with COVID-19 infection (*n* = 126)

Characteristics	Level	Hospitalization		
		*P* value	OR	95% CI
Sex	Male	0.014	5.967	(1.44, 24.71)
	Female*		1	
t-DMARDs	Yes	0.012	17.531	(1.85, 165.86)
	No*		1	
Glucocorticoids	Yes	0.014	6.692	(1.46, 30.64)
	No*		1	
Vaccinated	Yes	0.008	0.094	(0.02, 0.54)
	No*		1	

*t*-*DMARDs* targeted disease-modifying antirheumatic drugs

*Reference group

## Discussion

The present study is one of a few studies that investigated the clinical course of COVID-19 in children and adolescents with rheumatic diseases, and it is the first one to compare between the infected and non-infected patients in the same cohort, contributing to the global knowledge of COVID-19 in pediatric rheumatology.

This study was conducted over a 2-year period from March 2020 to March 2022. During this time, unnecessary hospital visits were restricted, and virtual clinics were implemented. Despite these measures, among 482 patients with rheumatic diseases who needed to visit our hospital, 26.1% (95% CI 21.8–31.1) contracted COVID-19 infection. One study done in Spain early through the pandemic included 959 patients with rheumatic diseases, reported a COVID-19 cumulative incidence of 1.21% (95% CI 0.42–1.99) compared to 0.58% (95% CI 0.56–0.60) in the general population. This study was done during a 2-week period (26 Mar–10 Apr, 2020), and no cases of COVID-19 were found among the pediatric rheumatology patients in their cohort [21].

In our study, 29.4% of the 126 patients with COVID-19 were asymptomatic, which is a similar percentage to what was found in the National Paediatric Rheumatology Database in Germany, where 18/76 patients had asymptomatic course [22]. A higher percentage was reported in Spain (30/77 asymptomatic patients) [23], whereas Haslak et al. found that only 13.4% of 658 pediatric rheumatology patients were asymptomatic during the time of infection [24]. This variability in the percentage of patients with asymptomatic course was reported also in healthy children with COVID-19. Souza et al. has reported in large meta-analysis study that 14.2% out of 1117 cases were asymptomatic [25], while Alharbi et al. found a higher percentage of asymptomatic patients in general pediatric population (54.6% out of 742) [11].

Nonetheless, most of the conducted studies among different cohorts (healthy individuals, patients with rheumatic diseases, adults, or pediatrics) found that fever is the most frequent reported symptom of COVID-19 followed by respiratory manifestations [9, 11, 22–26]. Our study findings concur with what was reported in previous studies.

Patients with rheumatic diseases have gained increased interest due to their susceptibility to infections. Since the beginning of the pandemic, several articles have been published rapidly attempting to investigate the effect of the virus on this population and to compare outcomes with those of a healthy population. In our study, the disease can be considered mild and self-limiting as most of the patients did not require hospitalization (85%). These reassuring findings regarding the disease course and outcome are in line with other reports in adult and pediatric patients with rheumatic diseases [6, 17, 22, 23]. However, given the limited number of studies and lacking the long-term prospective follow-up, data are still insufficient to draw a definite conclusion. Furthermore, one study showed a concerning result, where the presence of inflammatory rheumatic diseases was found to increase the risk of hospitalization and symptomatic infection [24]. This emphasizes the need for larger and prospective studies in this cohort of patients.

Data on investigating the differences between pediatric rheumatology patients who contracted COVID-19 infection and those who did not, looking for any risk factor that might increase the incidence of this infection are limited. Interestingly, there was no statistically significant association between gender, age groups, pre-existing comorbidities, COVID-19 vaccinations, nor using conventional or targeted DMARDs with the incidence of COVID-19 in our cohort.

Furthermore, this study explored the predictors of hospital admission due to COVID-19 in pediatric rheumatology patients. First, the risk of hospitalization in males was almost 6 times higher compared to females. This is like what was found in adults with rheumatic diseases [7] and European children and adolescents [26], where male sex was a significant risk factor for COVID-19-related hospitalization and ICU admission. Second, we found that vaccinated patients are 10 times less likely to be admitted due to COVID-19 in comparison with unvaccinated patients. This is supported by multiple randomized controlled studies, summarized in a recent systematic review [27].

With regard to medications used in pediatric rheumatology patients, the risk of hospitalization due to COVID-19 was higher in patients on glucocorticoids during the time of infection. This is in agreement with data reported in pediatrics and adults with rhematic diseases [7, 23, 28, 29]. In addition, several studies did not find a statistically significant association between DMARDs used (conventional or targeted) with COVID-19-related hospitalization [7, 8, 17, 21, 24, 29]. Our study is partially in agreement with these reports as using c-DMARDs was not associated with hospital admission due to COVID-19. However, ongoing t-DMARDs was found to increase the risk of COVID-19-related hospitalization in our cohort. Similarly, one study reported that using t-DMARDs during COVID-19 has an OR of 2.61 for hospitalization in patients with rheumatic diseases [30].

This study is the largest investigation of COVID-19 in patients (< 19 years) with rheumatic diseases in our region, reporting several important findings and contributing to the global knowledge of COVID19 in pediatric rheumatology. However, it has some limitations inherent in its retrospective design and being carried out in a single center, thus its results cannot be generalized considering the potential biases and the relatively small study sample. Furthermore, during the first half of the study period, a lockdown was in effect, and not all rheumatology cases presented to our hospital, thereby affecting the number of included subjects who had to continue their follow-ups virtually. In addition, laboratory investigations (other than COVID-19 PCR), medications’ dosages and other confounding factors were not collected from the patients’ files and, therefore, not accounted in the analysis.

## Conclusion

Understanding the clinical course of COVID-19 in children and adolescents with rheumatic diseases is crucial for all pediatric rheumatologists, especially when it comes to the predictors of severe infection requiring hospitalization. We studied a large cohort of pediatric rheumatology patients with one quarter of them had history of COVID-19 during the last 2 years. Most of the time, the course of illness is mild and self-limiting with no complications. However, our results suggest that being a male, ongoing t-DMARDs, and using glucocorticoids during the time of infection increase the risk of hospital admission, which may necessitate vigilant monitoring for those patients during the time of COVID-19 infection. Furthermore, we strongly advocate for the widespread promotion of COVID-19 vaccination among pediatric rheumatology patients as it significantly reduces their risk of COVID-19-related hospitalization. Finally, our study will be an important addition to the international literature regarding COVID-19, and, indeed, larger and prospective studies in this population are needed.

## Data Availability

All data are available upon request.

## Abbreviations

COVID-19: The coronavirus disease 2019
DMARDs: Disease-modifying antirheumatic drugs
c-DMARDs: Conventional disease-modifying antirheumatic drugs
t-DMARDs: Targeted disease-modifying antirheumatic drugs
ARDS: Acute respiratory distress syndrome
MIS-C: Multisystem inflammatory syndrome in children
PCR: Polymerase chain reaction
Anti-TNF: Tumor necrosis factor inhibitors
NSAIDs: Non-steroidal anti-inflammatory drugs
MMF: Mycophenolate mofetil

## References

[1] World Health Organization (2020) Novel coronavirus (2019-nCoV) SITUATION REPORT – 1. https://www.who.int/docs/default-source/coronaviruse/situation-reports/20200121-sitrep-1-2019ncov.pdf?sfvrsn=20a99c10.4. Accessed 15 May 2023

[2] Chen Y, Liu Q, Guo D (2020). Emerging coronaviruses: genome structure, replication, and pathogenesis. J Med Virol.

[3] Mehraeen E, Salehi MA, Behnezhad F, Moghaddam HR, SeyedAlinaghi S (2021). Transmission modes of COVID-19: a systematic review. Infect Disord Drug Targets.

[4] World Health Organization (2020) Coronavirus disease (COVID-19) Situation Report – 184. https://www.who.int/publications/m/item/weekly-epidemiological-update-on-covid-19---4-may-2023 Accessed 15 May 2023

[5] World Health Organization (2023) Statement on the fifteenth meeting of the IHR (2005) Emergency Committee on the COVID-19 pandemic. https://www.who.int/news/item/05-05-2023-statement-on-the-fifteenth-meeting-of-the-international-health-regulations-(2005)-emergency-committee-regarding-the-coronavirus-disease-(covid-19)-pandemic. Accessed 16 May 2023

[6] Monti S, Balduzzi S, Delvino P, Quadrelli VS, Montecucco C (2020). Clinical course of COVID-19 in a series of patients with chronic arthritis treated with immunosuppressive targeted therapies. Ann Rheumat Dis.

[7] Montero F, Martínez-Barrio J, Serrano-Benavente B, González T, Rivera J, Collada JM, Castrejón I, Álvaro-Gracia J (2020). Coronavirus disease 2019 (COVID-19) in autoimmune and inflammatory conditions: clinical characteristics of poor outcomes. Rheumatol Int.

[8] Alzahrani ZA, Alghamdi KA, Almaqati AS (2021). Clinical characteristics and outcome of COVID-19 in patients with rheumatic diseases. Rheumatol Int.

[9] Licciardi F, Giani T, Baldini L, Favalli EG, Caporali R, Cimaz R (2020). COVID-19 and what pediatric rheumatologists should know: a review from a highly affected country. Pediatr Rheumatol.

[10] Dong Y, Mo X, Hu Y, Qi X, Jiang F, Jiang Z, Tong S (2020). Epidemiology of COVID-19 among children in China. Pediatrics.

[11] Alharbi M, Kazzaz YM, Hameed T, Alqanatish J, Alkhalaf H, Alsadoon A, Alayed M, Hussien SA, Al Shaalan M, Al Johani SM (2021). SARS-CoV-2 infection in children, clinical characteristics, diagnostic findings and therapeutic interventions at a tertiary care center in Riyadh, Saudi Arabia. J Infect Public Health.

[12] Wu Z, McGoogan JM (2020). Characteristics of and important lessons from the coronavirus disease 2019 (COVID-19) outbreak in China: summary of a report of 72 314 cases from the Chinese Center for Disease Control and Prevention. JAMA.

[13] Hedrich CM (2020). COVID-19–considerations for the paediatric rheumatologist. Clin Immunol.

[14] Lee PI, Hu YL, Chen PY, Huang YC, Hsueh PR (2020). Are children less susceptible to COVID-19?. J Microbiol Immunol Infect.

[15] Penner J, Abdel-Mannan O, Grant K, Maillard S, Kucera F, Hassell J, Eyre M, Berger Z, Hacohen Y, Moshal K, Wyatt M (2021). 6-month multidisciplinary follow-up and outcomes of patients with paediatric inflammatory multisystem syndrome (PIMS-TS) at a UK tertiary paediatric hospital: a retrospective cohort study. Lancet Child Adolesc Health.

[16] Ouldali N, Toubiana J, Antona D, Javouhey E, Madhi F, Lorrot M, Léger PL, Galeotti C, Claude C, Wiedemann A, Lachaume N (2021). Association of intravenous immunoglobulins plus methylprednisolone vs immunoglobulins alone with course of fever in multisystem inflammatory syndrome in children. JAMA.

[17] Kearsley-Fleet L, Chang ML, Lawson-Tovey S, Costello R, Fingerhutová Š, Švestková N, Belot A, Aeschlimann FA, Melki I, Koné-Paut I, Eulert S (2022). Outcomes of SARS-CoV-2 infection among children and young people with pre-existing rheumatic and musculoskeletal diseases. Ann Rheum Dis.

[18] Kari JA, Shalaby MA, Albanna AS, Alahmadi TS, Sukkar SA, Mohamed Nur HA, AlGhamdi MS, Basri AH, Shagal RA, Alnajar A, Badawi M (2021). Coronavirus disease in children: a multicentre study from the Kingdom of Saudi Arabia. J Infect Public Health.

[19] Al-Harbi S, Kazzaz YM, Uddin MS, Maghrabi F, Alnajjar AA, Muzaffer M, Alnahdi BM, Abusaif SM, Alhejaili A, Aljohnei R, Arishi S (2021). Clinical characteristics and outcomes of multisystem inflammatory syndrome in children (MIS-C): a national multicenter cohort in Saudi Arabia. Curr Pediatr Res.

[20] Centers for Disease Control and Prevention (2020) Information for healthcare providers about multisystem inflammatory syndrome in children (MIS-C). https://www.cdc.gov/mis-c/hcp/. Accessed 20 May 2023

[21] Michelena X, Borrell H, López-Corbeto M, López-Lasanta M, Moreno E, Pascual-Pastor M, Erra A, Serrat M, Espartal E, Antón S, Añez GA (2020). Incidence of COVID-19 in a cohort of adult and paediatric patients with rheumatic diseases treated with targeted biologic and synthetic disease-modifying anti-rheumatic drugs. Semin Arthritis Rheum.

[22] Sengler C, Eulert S, Minden K, Niewerth M, Horneff G, Kuemmerle-Deschner J, Siemer C, Berendes R, Girschick H, Hühn R, Borte M (2021). Clinical manifestations and outcome of SARS-CoV-2 infections in children and adolescents with rheumatic musculoskeletal diseases: data from the National Paediatric Rheumatology Database in Germany. RMD Open.

[23] Clemente D, Udaondo C, De Inocencio J, Nieto JC, Del Río PG, Fernández AG, Palomo JA, Bachiller-Corral J, Lopez Robledillo JC, Millán Longo C, Leon L (2021). Clinical characteristics and COVID-19 outcomes in a regional cohort of pediatric patients with rheumatic diseases. Pediatr Rheumatol.

[24] Haslak F, Varol SE, Gunalp A, Kaynar O, Yildiz M, Adrovic A, Sahin S, Kes G, Ayzit-Kilinc A, Akdeniz B, Onal P (2022). Comparisons of clinical features and outcomes of COVID-19 between patients with pediatric onset inflammatory rheumatic diseases and healthy children. J Clin Med.

[25] De Souza TH, Nadal JA, Nogueira RJ, Pereira RM, Brandão MB (2020). Clinical manifestations of children with COVID-19: a systematic review. Pediatr Pulmonol.

[26] Götzinger F, Santiago-García B, Noguera-Julián A, Lanaspa M, Lancella L, Calò Carducci FI (2020). COVID-19 in children and adolescents in Europe: a multinational, multicentre cohort study. Lancet Child Adolesc Heal.

[27] Graña C, Ghosn L, Evrenoglou T, Jarde A, Minozzi S (2022). Efficacy and safety of COVID-19 vaccines. Cochrane Database Syst Rev.

[28] Freites Nuñez DD, Leon L, Mucientes A, Rodriguez-Rodriguez L, Font Urgelles J, Madrid García A (2020). Risk factors for hospital admissions related to COVID-19 in patients with autoimmune inflammatory rheumatic diseases. Ann Rheum Dis.

[29] Gianfrancesco M, Hyrich KL, Hyrich KL, Al-Adely S, Al-Adely S, Carmona L (2020). Characteristics associated with hospitalisation for COVID-19 in people with rheumatic disease: data from the COVID-19 global rheumatology alliance physician-reported registry. Ann Rheum Dis.

[30] Jovani V, Calabuig I, Peral-Garrido ML, Tovar-Sugrañes E, López-González MD, Bernabeu P (2022). Incidence of severe COVID-19 in a Spanish cohort of 1037 patients with rheumatic diseases treated with biologics and JAK-inhibitors. Ann Rheum Dis.

